# Hormone Replacement Cycle Frozen–Thawed Embryo Transfer Is Associated With Elevated Perinatal Risk Compared With Natural Ovulatory Cycle Frozen–Thawed and Fresh Embryo Transfers: Retrospective Analysis of 7,593 Live Birth Cycles

**DOI:** 10.1002/rmb2.70072

**Published:** 2026-07-06

**Authors:** Satoko Fujioka, Hiroshi Matsumoto, Haruhisa Konishi, Naoko Terawaki, Yoshiharu Nakaoka, Aisaku Fukuda, Yoshiharu Morimoto

**Affiliations:** ^1^ IVF Osaka Clinic Higashi‐Osaka City Osaka Japan; ^2^ IVF Namba Clinic Naniwa‐ku Osaka Japan; ^3^ HORAC Grand Front Osaka Clinic Kita‐ku Osaka Japan

**Keywords:** fresh embryo transfer, frozen–thawed embryo transfer, hormone replacement cycle, natural cycle, perinatal complications

## Abstract

**Purpose:**

This study investigated whether perinatal complications in assisted reproductive technology are attributable to embryo cryopreservation or the specific endometrial preparation method, comparing Fresh Embryo Transfer (Fresh ET), Natural Cycle Frozen–thawed Embryo Transfer (NC‐FET), and Hormone Replacement Cycle FET (HRC‐FET).

**Methods:**

A retrospective cohort analysis of 7,593 live‐birth cycles (908 Fresh ET, 4,707 HRC‐FET, 1,978 NC‐FET) performed from 2015 to 2023 was conducted. Multivariable analyses using generalized estimating equations, adjusted for endometrial preparation method, maternal age, BMI, history of delivery, and endometrial thickness, were conducted for the five major complications.

**Results:**

Compared with Fresh ET, HRC‐FET was associated with significantly increased risks for hypertensive disorders of pregnancy, placenta accreta spectrum, and postpartum hemorrhage due to uterine atony. No significantly increased risks for these adverse outcomes were observed in association with NC‐FET. No significant differences were observed for gestational diabetes or placenta previa across groups.

**Conclusions:**

The increased risks of adverse maternal and placental outcomes were associated with the HRC protocol rather than the cryopreservation process. These associations suggest that ovulatory‐based endometrial preparation strategies, typically characterized by the presence of a corpus luteum, should be considered when medically and logistically feasible to enhance perinatal safety.

## 1 Introduction

In Japan, the initiation of public health insurance coverage for infertility treatment utilizing Assisted Reproductive Technology (ART) in April 2022 precipitated a marked surge in procedural volume, with the number of ART cycles escalating to 561,664 in 2023 [1, 2]. The resulting number of ART‐derived live births totaled 85,048. Of these births, frozen–thawed embryo transfer (FET) accounted for a vast majority, 80 774 individuals, or 95%, while fresh embryo transfers (Fresh ET) accounted for only 4 274 births, corresponding to 5%.

The predominance of FET is fundamentally linked to advancements in cryopreservation technology. FET is no longer confined to merely utilizing supernumerary embryos following a fresh transfer cycle. It has transitioned into a primary therapeutic strategy, utilized with the aims of avoiding ovarian hyperstimulation syndrome (OHSS) [3, 4] and enhancing overall pregnancy rates [5, 6]. Furthermore, the proliferation of controlled ovarian stimulation protocols such as progestin‐primed ovarian stimulation (PPOS) has integrated total embryo freezing as an essential component of the treatment regimen.

However, ART pregnancies are consistently reported to carry a significantly elevated risk of various perinatal complications when compared to spontaneous conception [7, 8, 9, 10]. These complications include, but are not restricted to, hypertensive disorders of pregnancy (HDP), gestational diabetes mellitus (GDM), placenta previa, placenta accreta spectrum (PAS), and postpartum hemorrhage (PPH). When comparing ART protocols, FET has been specifically reported to be associated with a higher risk of perinatal complications than Fresh ET [11, 12, 13, 14]. Delving further into FET protocols, HRC‐FET has been noted to confer greater risks for certain perinatal complications, such as PAS and HDP, compared to NC‐FET [15, 16, 17, 18].

The incidence of these perinatal complications is multifactorial, known to correlate with diverse maternal and procedural elements, including maternal age [19, 20], maternal body mass index (BMI) [21, 22, 23], parity [24, 25], endometrial thickness at the time of embryo transfer [26, 27], gestational age [28, 29, 30], multiple gestations [31, 32, 33], and the mode of delivery [34]. While HRC‐FET currently represents the mainstream approach for endometrial preparation, there has been a recent impetus to re‐evaluate Fresh ET and NC‐FET as strategies potentially mitigating the risk of perinatal complications.

Crucially, previous large‐scale investigations into the relative risk of perinatal complications associated with transfer methods have predominantly relied upon two‐group comparisons (e.g., Fresh ET versus FET, or NC‐FET versus HRC‐FET). Consequently, prior research has left ambiguous whether the observed complication risk is attributable to the intrinsic effect of embryo cryopreservation itself or whether it is primarily mediated by the specific endometrial preparation method employed during the transfer cycle.

In addressing this critical gap, we investigated the occurrence of perinatal complications in live‐birth cycles resulting from three distinct embryo transfer protocols, including Fresh ET, NC‐FET, and HRC‐FET. Our primary objective was to rigorously examine whether a specific embryo transfer method is associated with differences in perinatal complication risk, incorporating careful consideration of confounding factors.

## 2 Materials and Methods

### 2.1 Study Design and Population

This retrospective cohort study analyzed a large number of embryo transfer cycles resulting in live births, conducted between 2015 and 2023 across multiple facilities including IVF Osaka Clinic, IVF Namba Clinic, and HORAC Grand Front Osaka Clinic. The cycles were specifically categorized into three distinct endometrial preparation methods: Fresh ET cycles, FET performed in HRC, and FET performed in NC. The study cohort comprised 908 Fresh ET cycles, 4707 HRC‐FET, and 1978 NC‐FET.

### 2.2 Oocyte Retrieval and IVF

Ovarian stimulation was performed according to each patient's medical history, in accordance with previously described protocols [35]. Briefly, the patients received oral contraceptives for 10 days (Planovar, Aska, Tokyo, Japan) starting from day 14 of the previous menstrual cycle. Beginning on day 3 of the cycle, 150–300 IU of follicle‐stimulating hormone (FSH) (Gonalef, Merck, Tokyo, Japan) was administered for four consecutive days. This was followed by administration of 150–450 IU of urinary human menopausal gonadotropin (HMG) (HMG for injection, Ferring, Tokyo, Japan), followed by human chorionic gonadotropin (hCG) (Ovidrel, Merck, Tokyo, Japan) administration. In the gonadotropin‐releasing hormone (GnRH) agonist long protocol, GnRH agonist (Suprecur, Sanofi, Tokyo, Japan; Buserelin Nasal Solution, Fuji Pharma, Tokyo Japan) was started from day 21 of the previous cycle and continued until hCG administration. In the short protocol, GnRH agonist was given from day 2 of the cycle, followed by hCG administration. In the GnRH antagonist protocol, once follicles reached 14 mm in diameter, once‐daily GnRH antagonist (Cetrotide, Nippon Kayaku, Tokyo, Japan; Ganirest, MSD, Tokyo, Japan; or Relumina, Aska, Tokyo, Japan) was administered until hCG administration began. In the PPOS protocol, medroxyprogesterone acetate (Medroxyprogesterone Acetate Tablets, Fuji Pharma, Tokyo, Japan) at 10 mg/day or dydrogesterone (Duphaston, Viatris, Tokyo, Japan) at 20 mg/day was administered until hCG administration. In the PPOS protocol, all embryos were cryopreserved. In the mild stimulation protocol, clomiphene citrate (Clomid, Fuji Pharma, Tokyo, Japan) or 150 IU of HMG was administered from day 3 of the cycle until hCG administration. Once‐daily GnRH antagonist administration was initiated after the follicle diameter reached 14 mm. When the dominating follicle reached 18 mm in diameter, hCG was administered to induce ovulation. Transvaginal oocyte retrieval was carried out 36 h after the trigger. The collected cumulus‐oocyte complexes were cultured until fertilization. Fertilization was accomplished by either conventional IVF or intracytoplasmic sperm injection. Oocytes exhibiting two pronuclei 16–20 h post‐fertilization were deemed normally fertilized. These embryos were subsequently cultured until day 2, day 3, or day 5, at which point morphological assessment was performed to determine suitability for embryo transfer.

### 2.3 Embryo Cryopreservation and Thawing

In some cases, embryos were cryopreserved to prevent OHSS or to facilitate the subsequent utilization of surplus embryos. The vitrification method was used for cryopreservation, employing the vitrification kit (Cryotop Safety Kit, Kitazato Corp., Shizuoka, Japan) according to the manufacturer's guidelines. These cryopreserved embryos were thawed and transferred in a subsequent cycle, with the thawing process performed using the thawing kit (Cryotop Safety Kit, Kitazato Corp., Shizuoka, Japan) as per the manufacturer's instructions.

### 2.4 Embryo Transfer

ET was performed during either fresh or frozen–thawed cycles. For fresh cycles, embryos were transferred 2 or 3 days (cleavage stage) or 5 days (blastocysts) after oocyte retrieval in patients with an endometrial thickness > 8 mm on the day of hCG injection. Daily progesterone doses of 90 mg (OneCrinone, Merck, Tokyo, Japan) were maintained until 9 weeks of gestation. In frozen–thawed cycles, the endometrium was prepared in two ways. In hormone replacement cycles, incremental doses of oral estradiol (Estradiol Tablets, Fuji Pharma, Tokyo, Japan) ranging from 1 to 4 mg over 2 weeks, following 3 weeks of daily GnRH agonist administration. After confirming an endometrial thickness of > 8 mm by ultrasound, 600 mg of progesterone (Utrogestan, Fuji Pharma, Tokyo, Japan) was administered daily. Frozen–thawed embryos were transferred on the second/third or fifth day of progesterone administration. Daily doses of 4 mg of estradiol and 600 mg of progesterone were maintained until 9 weeks of gestation. In natural cycles, follicular development was followed by ultrasound. Once follicles reached 18 mm, human chorionic gonadotropin was administered and also LH was measured to set embryo transfer. When LH was < 15 IU/L, ovulation was supposed to occur 2 days later. Whereas, when LH was 15 IU/L or higher, ovulation was supposed to occur the next day. In both cases, 600 mg of progesterone was administered for 2 or 3 days or 5 days from the estimated ovulation date. Frozen–thawed embryos were transferred on the second/third or fifth day of progesterone administration. Daily doses of 600 mg of progesterone were maintained until 9 weeks of gestation.

### 2.5 Data Collection

A complete‐case analysis was performed for this study. Cases with missing data for any of the analyzed variables were excluded from the final cohort. The proportion of missing values for each covariate was less than 2.5%; therefore, no imputation methods were employed. Baseline demographic and clinical characteristics of patients and their partners were systematically collected for each ET cycle. These included maternal age at transfer, husband's age at transfer, maternal BMI, history of delivery, etiologies of infertility, and endometrial thickness at transfer. Furthermore, perinatal clinical characteristics and complications following successful embryo transfer were recorded. These included maternal age at delivery, mode of delivery (vaginal delivery or cesarean section), incidence of multiple birth, gestational age at delivery, perinatal complications, specifically HDP, GDM, placenta previa, PAS, and PPH due to uterine atony. Composite outcomes, including any of the five specified complications and multiple complications among the five, were also evaluated.

### 2.6 Statistical Analysis

Baseline characteristics and perinatal outcomes across the three study groups were compared using the Kruskal–Wallis test for continuous variables and the chi‐squared test for categorical variables. For post hoc comparisons of continuous variables, the Mann–Whitney U test with Bonferroni correction was used. Categorical variables were further analyzed using chi‐squared tests with Bonferroni correction to account for multiple comparisons. Regarding baseline characteristics of patients and ET, we assessed group comparability and balance in this large observational cohort primarily by using standardized mean differences (SMDs) as the indicator of imbalance, with conventional hypothesis tests serving as a supplementary assessment. This approach was adopted because *p*‐values in large datasets can highlight statistically significant but clinically trivial differences, whereas SMDs provide a scale‐invariant measure of the magnitude of divergence between groups. An SMD exceeding 0.1 was interpreted as reflecting a meaningful imbalance. Regarding perinatal outcomes, the comparison was designed to be primarily descriptive, focusing on the quantification of clinical relevance. To this end, we emphasized effect sizes, reported as mean differences (MDs) for continuous variables and risk differences (RDs) for categorical variables, each accompanied by its respective 95% CIs. Both MD and RD were defined as the values in the exposed groups (HRC‐FET or NC‐FET) minus those in the reference group (Fresh ET), such that positive values indicate higher means or increased risks in the FET groups. Unadjusted *p*‐values from Kruskal–Wallis and chi‐squared tests were interpreted cautiously in the context of the observed effect magnitudes.

Furthermore, we performed multivariable analysis to estimate the causal effect of endometrial preparation methods on perinatal complications. To achieve this, we employed Generalized Estimating Equation (GEE) models with logit functions, accounting for multiple cycles per patient. Based on the conceptual framework of causal inference, our primary model (total effect model) adjusted only for pre‐exposure confounding factors, including maternal age at transfer, BMI, history of delivery, and endometrial thickness at transfer. Fresh ET was designated as the reference group to compare the risks associated with HRC‐FET and NC‐FET.

To verify the robustness of causal estimates, we conducted two additional sensitivity analyses. First, we performed stratified analyses by maternal age, categorizing the study population into two groups (< 36 and > 35 years). Second, we performed a direct comparison between NC‐FET and HRC‐FET protocols within the FET cohort, with NC‐FET serving as the reference group, to isolate the impact of the endometrial preparation method from the cryopreservation process. All continuous variables were modeled as linear terms. A *p* < 0.05 was considered statistically significant. All statistical analyses were performed using EZR software, version 1.68 [36].

## 3 Results

### 3.1 Characteristics of Patients and Embryo Transfer

The baseline characteristics of patients and ET, as well as the SMDs between the groups, were summarized in Table 1. Regarding parental age, maternal age at transfer was lower in the Fresh ET group compared to the FET groups, with SMDs exceeding the 0.1 threshold (Fresh vs. HRC: 0.119, Fresh vs. NC: 0.121). This difference was also statistically significant (*p* < 0.001). A similar pattern of imbalance was observed for husband's age (SMD: 0.128 for Fresh vs. HRC and 0.130 for Fresh vs. NC; *p* = 0.005). Maternal BMI was higher in the Fresh ET group, reflecting a meaningful imbalance compared to the HRC‐FET and NC‐FET groups (SMD 0.128 and 0.183, respectively). This difference reached statistical significance (*p* = 0.001). In terms of obstetric history, the proportion of patients with a history of delivery was lower in the Fresh ET group, showing a meaningful imbalance between groups (SMD: 0.256 for Fresh vs. HRC and 0.273 for Fresh vs. NC). This demographic difference was also statistically significant (*p* < 0.001). Analysis of infertility etiologies showed that factors such as thyroid dysfunction, tubal factor, myoma, endometrioma, hyperprolactinemia, and adenomyosis were well‐balanced across all three groups (all SMDs < 0.1). Male factor infertility was balanced between the Fresh ET and HRC‐FET groups (SMD < 0.1) while a marginal imbalance was shown between the Fresh ET and NC‐FET groups (SMD 0.104). This difference was not statistically significant. The prevalence of aging‐related infertility was lower in the Fresh ET group (SMD: 0.149 for Fresh vs. HRC and 0.128 for Fresh vs. NC; *p* < 0.001), while obesity was more prevalent (SMD: 0.118 for Fresh vs. HRC and 0.143 for Fresh vs. NC; *p* < 0.001). The most pronounced imbalance was observed in endometrial thickness at the time of transfer, which was greater in the Fresh ET group compared to the HRC‐FET and NC‐FET groups (SMD 0.364 and 0.322, respectively; *p* < 0.001).

**TABLE 1:** Baseline characteristics of patients and embryo transfer.

Characteristic	Fresh ET	HRC‐FET	NC‐FET	SMD	SMD	*p*
	(*n* = 908)	(*n* = 4707)	(*n* = 1978)	(Fresh vs HRC)	(Fresh vs. NC)	
Maternal age at transfer (year)	35.9 ± 3.8^a^	36.4 ± 4.0^b^	36.4 ± 4.0^b^	0.119	0.121	< 0.001
Husband's age at transfer (year)	37.8 ± 5.3^a^	38.5 ± 5.5^b^	38.5 ± 5.6^b^	0.128	0.130	0.005
BMI (kg/m^2^)	21.4 ± 3.3^a^	21.0 ± 2.9^b^	20.8 ± 2.8^b^	0.128	0.183	0.001
History of delivery	174 (19.2)^a^	1416 (30.1)^b^	611 (30.9)^b^	0.256	0.273	< 0.001
Etiology of infertility*						
Male**	649 (71.5)	3510 (74.6)	1469 (75.3)	0.070	0.104	0.147
Thyroid dysfunction	211 (23.2)	1143 (24.3)	442 (22.3)	0.025	0.001	0.224
Tubal	194 (21.4)	1012 (21.5)	400 (20.2)	0.003	0.093	0.498
Aging	141 (15.5)^a^	1002 (21.3)^b^	404 (20.4)^b^	0.149	0.128	< 0.001
Myoma	162 (17.8)	898 (19.1)	375 (19.0)	0.032	0.092	0.682
Endometrioma	141 (15.5)	668 (14.2)	302 (15.3)	0.038	0.007	0.376
Hyperprolactinemia	58 (6.4)	348 (7.4)	132 (6.7)	0.04	0.012	0.395
Obesity	57 (6.3)^a^	175 (3.7)^b^	64 (3.2)^b^	0.118	0.143	< 0.001
Adenomyosis	20 (2.2)	103 (2.2)	44 (2.2)	0.001	0.001	0.996
Endometrial thickness at transfer (mm)	11.9 ± 2.2^a^	11.1 ± 1.9^b^	11.2 ± 1.9^b^	0.364	0.322	< 0.001

*Note:* Data were presented as mean ± SD or *n* (%). Different superscript letters in the same row indicate a statistically significant difference in the post hoc test.

Abbreviations: BMI, body mass index; FET, frozen–thawed embryo transfer; HRC, hormone replacement cycle; NC, natural cycle; SMD, standardized mean difference.

* Infertility etiologies are not mutually exclusive; each category encompasses both isolated and mixed factors.

** Male factor was defined according to institutional criteria (semen volume < 2.0 mL, sperm concentration < 75 × 10^6^/mL, motility < 50%, or normal morphology < 65%), which are higher than standard WHO thresholds.

### 3.2 Perinatal Clinical Characteristics and Descriptive Comparisons

The perinatal clinical characteristics and descriptive comparisons across the three groups were summarized in Table 2. While maternal age at delivery demonstrated a statistically significant difference, the actual magnitude of this difference was marginal for both FET groups. The MD versus Fresh ET was 0.46 years (95% CI: 0.18 to 0.73) for HRC‐FET and 0.46 years (95% CI: 0.16 to 0.77) for NC‐FET, indicating that the differences in maternal age were small and unlikely to be clinically meaningful. Similarly, gestational age at delivery showed minimal MDs, such as 0.08 weeks (95% CI: −0.05 to 0.21) for HRC‐FET and −0.11 weeks (95% CI: −0.26 to 0.03) for NC‐FET, suggesting that the differences were minimal and unlikely to be clinically meaningful. In terms of clinical observations, substantial variations were noted in the mode of delivery. The HRC‐FET group was characterized by a lower rate of vaginal delivery (54.7%) compared to Fresh ET (70.9%), with an RD of −0.16 (95% CI: −0.19 to −0.12). While the NC‐FET group also showed a lower proportion of vaginal deliveries (62.9%), the RD was less pronounced at −0.08 (95% CI: −0.11 to −0.04). The incidence of multiple births remained low across all three cohorts (2.6% to 3.5%), with minimal RDs compared to Fresh ET (RD: 0.005 for HRC‐FET and 0.008 for NC‐FET).

**TABLE 2:** Perinatal clinical data and descriptive comparisons across groups.

Characteristic	Fresh ET	HRC‐FET	NC‐FET	MD, RD (Fresh vs HRC)	MD, RD (Fresh vs NC)	
	(*n* = 908)	(*n* = 4707)	(*n* = 1978)	[95% CI]	[95% CI]	*p* *
Maternal age at delivery (year)	36.7 ± 3.7^a^	37.1 ± 4.0^b^	37.1 ± 4.0^b^	MD: 0.46 [0.18 to 0.73]	MD: 0.46 [0.16 to 0.77]	0.003
Mode of delivery						
Vaginal delivery	644 (70.9)^a^	2577 (54.7)^b^	1245 (62.9)^c^	RD: −0.16 [−0.19 to −0.12]	RD: −0.08 [−0.11 to −0.04]	< 0.001
Cesarean section	264 (29.1)	2130 (45.3)	733 (37.1)			
Multiple birth	24 (2.6)	148 (3.1)	69 (3.5)	RD: 0.005 [−0.007 to 0.017]	RD: 0.008 [−0.005 to 0.022]	0.477
Gestational age at delivery (week)	38.9 ± 1.8^ab^	39.0 ± 2.0^a^	38.8 ± 1.8^b^	MD: 0.08 [−0.05 to 0.21]	MD: −0.11 [−0.26 to 0.03]	< 0.001
Perinatal complications						
HDP	58 (6.4)^a^	504 (10.7)^b^	111 (5.6)^a^	RD: 0.04 [0.02 to 0.06]	RD: −0.008 [−0.02 to 0.01]	< 0.001
GDM	55 (6.1)	276 (5.9)	97 (4.9)	RD: −0.002 [−0.019 to 0.015]	RD: −0.012 [−0.03 to 0.007]	0.252
Placenta previa	10 (1.1)	90 (1.9)	35 (1.8)	RD: 0.008 [0.0003 to 0.016]	RD: 0.007 [−0.002 to 0.016]	0.239
PAS	9 (1.0)^a^	246 (5.2)^b^	33 (1.7)^a^	RD: 0.04 [0.03 to 0.05]	RD: 0.007 [−0.002 to 0.015]	< 0.001
PPH due to uterine atony	27 (3.0)^a^	430 (9.1)^b^	79 (4.0)^a^	RD: 0.06 [0.04 to 0.07]	RD: 0.01 [−0.004 to 0.024]	< 0.001
Any of the five complications	145 (16.0)^a^	1311 (27.9)^b^	324 (16.4)^a^	RD: 0.11 [0.09 to 0.14]	RD: 0.004 [−0.025 to 0.033]	< 0.001
Multiple complications among the five	14 (1.5)^a^	211 (4.5)^b^	29 (1.5)^a^	RD: 0.02 [0.01 to 0.03]	RD: −0.001 [−0.01 to 0.009]	< 0.001

*Note:* Data were presented as mean ± SD or *n* (%). Different superscript letters in the same row indicate a statistically significant difference in the post hoc test. MD/RD were calculated as (FET groups—Fresh ET), such that positive values indicate higher means or increased risks in the FET groups.

Abbreviations: CI, confidence interval; FET, frozen–thawed embryo transfer; GDM, gestational diabetes mellitus; HDP, hypertensive disorders of pregnancy; HRC, hormone replacement cycle; MD, mean difference; NC, natural cycle; PAS, placenta accreta spectrum; PPH, postpartum hemorrhage; RD, risk difference.

* Unadjusted *p*‐values from Kruskal–Wallis or Chi‐square tests.

Regarding perinatal complications, the HRC‐FET protocol demonstrated a higher incidence of several key morbidities. Specifically, the RD for HDP was 0.04 (95% CI: 0.02 to 0.06), for PAS was 0.04 (95% CI: 0.03 to 0.05), and for PPH due to uterine atony was 0.06 (95% CI: 0.04 to 0.07), reflecting a clinically relevant elevation in risk compared to Fresh ET. In contrast, the NC‐FET group showed no such risk elevation, with RDs for HDP, PAS, and PPH being −0.008 (95% CI: −0.02 to 0.01), 0.007 (95% CI: −0.002 to 0.015), and 0.01 (95% CI: −0.004 to 0.024), respectively, with all 95% CIs crossing zero. Furthermore, the risks for GDM and Placenta previa appeared similar across all groups, with negligible RDs. The aggregate risk for any of the five complications was higher only in the HRC‐FET group (RD: 0.11, 95% CI: 0.09 to 0.14), while the NC‐FET group (RD: 0.004, 95% CI: −0.025 to 0.033) showed a risk similar to that of Fresh ET. Similarly, multiple complications were more prevalent in the HRC‐FET group (RD: 0.02, 95% CI: 0.01 to 0.03), whereas the risk in the NC‐FET group (RD: −0.001, 95% CI: −0.01 to 0.009) was similar to that of Fresh ET.

### 3.3 Multivariable Analysis of Perinatal Complications

The results of the multivariable analysis, which adjusted for pre‐exposure confounders to estimate the total effect of endometrial preparation methods, are shown in Table 3. Compared with Fresh ET, HRC‐FET was associated with significantly higher odds of several major complications, including HDP (aOR 1.96; 95% CI, 1.46 to 2.62), PAS (aOR 4.71; 95% CI, 2.41 to 9.21), and PPH due to uterine atony (aOR 3.29; 95% CI, 2.21 to 4.90). Regarding composite outcomes, HRC‐FET was also associated with a higher frequency of any of the five complications (aOR 2.09; 95% CI, 1.72 to 2.53) and multiple complications (aOR 3.05; 95% CI, 1.77 to 5.25). In contrast to the findings for HRC‐FET, NC‐FET showed no statistically significant differences in the risk of any perinatal complication relative to Fresh ET. Additionally, no significant associations were observed for GDM or placenta previa across any of the endometrial preparation methods compared to Fresh ET.

**TABLE 3:** Association between endometrial preparation methods and the risk of perinatal complications.

Complication	Method	aOR (95% CI)	*p* for aOR
HDP	Fresh ET	Reference	
	HRC‐FET	1.96 (1.46 to 2.62)	< 0.001
	NC‐FET	0.983 (0.702 to 1.37)	0.922
GDM	Fresh ET	Reference	
	HRC‐FET	0.955 (0.702 to 1.29)	0.771
	NC‐FET	0.809 (0.571 to 1.14)	0.236
Placenta previa	Fresh ET	Reference	
	HRC‐FET	1.69 (0.881 to 3.25)	0.113
	NC‐FET	1.57 (0.772 to 3.21)	0.211
PAS	Fresh ET	Reference	
	HRC‐FET	4.71 (2.41 to 9.21)	< 0.001
	NC‐FET	1.45 (0.695 to 3.05)	0.318
PPH due to uterine atony	Fresh ET	Reference	
	HRC‐FET	3.29 (2.21 to 4.90)	< 0.001
	NC‐FET	1.37 (0.878 to 2.13)	0.164
Any of the five complications	Fresh ET	Reference	
	HRC‐FET	2.09 (1.72 to 2.53)	< 0.001
	NC‐FET	1.06 (0.860 to 1.32)	0.545
Multiple complications among the five	Fresh ET	Reference	
	HRC‐FET	3.05 (1.77 to 5.25)	< 0.001
	NC‐FET	0.986 (0.519 to 1.87)	0.966

*Note:* The covariates for multivariable analysis included endometrial preparation methods, maternal age at transfer, BMI, history of delivery, and endometrial thickness at transfer.

Abbreviations: aOR, adjusted odds ratio; BMI, body mass index; CI, confidence interval; FET, frozen–thawed embryo transfer; GDM, gestational diabetes mellitus; HDP, hypertensive disorders of pregnancy; HRC, hormone replacement cycle; NC, natural cycle; PAS, placenta accreta spectrum; PH, postpartum hemorrhage.

To verify the robustness of our primary findings, we conducted stratified and direct comparison analyses as sensitivity tests (Table 4). The increased risks associated with HRC‐FET for HDP, PAS, and PPH due to uterine atony remained evident across both age groups (< 36 and > 35 years). Specifically, for HRC‐FET compared with Fresh ET, the aORs in the < 36 years and > 35 years strata were 2.11 (95% CI, 1.31 to 3.39) and 1.83 (95% CI, 1.26 to 2.66) for HDP, 21.0 (95% CI, 2.93 to 150) and 2.71 (95% CI, 1.32 to 5.59) for PAS, and 3.55 (95% CI, 2.00 to 6.29) and 3.04 (95% CI, 1.75 to 5.28) for PPH due to uterine atony, respectively. Regarding composite outcomes, HRC‐FET was associated with significantly higher odds of any of the five complications (aOR 3.05 in < 36 years; aOR 1.60 in > 35 years) and multiple complications (aOR 2.35 in < 36 years; aOR 3.71 in > 35 years) across both age groups. Regarding NC‐FET, no significant associations were observed for any of the individual complications or composite outcomes in either age stratum relative to Fresh ET.

**TABLE 4:** Sensitivity analyses for perinatal complications: Maternal age‐stratified analysis and direct comparison of frozen–thawed embryo transfer protocols.

		Maternal age < 36	Maternal age > 35	Direct comparison (FET only)
Complication	Method	aOR (95% CI)	aOR (95% CI)	aOR (95% CI)
HDP	Fresh ET	Reference	Reference	NA
	HRC‐FET	2.11 (1.31 to 3.39)	1.83 (1.26 to 2.66)	1.99 (1.61 to 2.47)
	NC‐FET	1.08 (0.626 to 1.86)	0.906 (0.591 to 1.38)	Reference
GDM	Fresh ET	Reference	Reference	NA
	HRC‐FET	1.24 (0.710 to 2.17)	0.852 (0.589 to 1.23)	1.18 (0.928 to 1.50)
	NC‐FET	0.873 (0.443 to 1.72)	0.774 (0.513 to 1.16)	Reference
Placenta previa	Fresh ET	Reference	Reference	NA
	HRC‐FET	2.48 (0.762 to 8.08)	1.35 (0.614 to 2.97)	1.07 (0.723 to 1.58)
	NC‐FET	2.24 (0.634 to 7.95)	1.27 (0.537 to 3.01)	Reference
PAS	Fresh ET	Reference	Reference	NA
	HRC‐FET	21.0 (2.93 to 150)	2.71 (1.32 to 5.59)	3.23 (2.24 to 4.66)
	NC‐FET	6.00 (0.785 to 45.8)	0.895 (0.393 to 2.03)	Reference
PPH due to uterine atony	Fresh ET	Reference	Reference	NA
	HRC‐FET	3.55 (2.00 to 6.29)	3.04 (1.75 to 5.28)	2.41 (1.88 to 3.08)
	NC‐FET	1.62 (0.859 to 3.08)	1.15 (0.619 to 2.14)	Reference
Any of the five complications	Fresh ET	Reference	Reference	NA
	HRC‐FET	3.05 (2.21 to 4.21)	1.60 (1.25 to 2.04)	1.95 (1.70 to 2.24)
	NC‐FET	1.41 (0.981 to 2.03)	0.871 (0.663 to 1.14)	Reference
Multiple complications among the five	Fresh ET	Reference	Reference	NA
	HRC‐FET	2.35 (1.08 to 5.10)	3.71 (1.72 to 8.00)	3.09 (2.09 to 4.58)
	NC‐FET	0.960 (0.374 to 2.46)	1.03 (0.427 to 2.50)	Reference

*Note:* The covariates for multivariable analysis included endometrial preparation methods, maternal age at transfer, BMI, history of delivery, and endometrial thickness at transfer.

Abbreviations: aOR, adjusted odds ratio; BMI, body mass index; CI, confidence interval; FET, frozen–thawed embryo transfer; GDM, gestational diabetes mellitus; HDP, hypertensive disorders of pregnancy; HRC, hormone replacement cycle; NC, natural cycle; PAS, placenta accreta spectrum; PPH, postpartum hemorrhage.

In a direct comparison within the FET cohort (using NC‐FET as the reference), HRC‐FET was associated with a significantly higher risk of complications, including HDP (aOR 1.99; 95% CI, 1.61 to 2.47), PAS (aOR 3.23; 95% CI, 2.24 to 4.66), and PPH due to uterine atony (aOR 2.41; 95% CI, 1.88 to 3.08) compared to NC‐FET. Regarding composite outcomes, HRC‐FET was also associated with a significantly higher risk of any of the five complications (aOR 1.95; 95% CI, 1.70 to 2.24) and multiple complications (aOR 3.09; 95% CI, 2.09 to 4.58). The aORs and 95% CIs for all covariates in the primary model and sensitivity analyses were presented in Tables S1–, S7.

## 4 Discussion

In the present study, we examined the relationship between specific endometrial preparation protocols in ART and the incidence of severe perinatal complications. Our primary analysis, focused on estimating the total causal effect, demonstrated that HRC‐FET is associated with significantly elevated risks of major maternal and placental morbidities, specifically HDP, PAS, and PPH due to uterine atony. The robustness of these findings was further validated through sensitivity analyses. Stratified analyses by maternal age revealed that the increased risks associated with HRC‐FET remained evident across both younger and older cohorts. A key insight from our investigation is provided by the direct comparison between NC‐FET and HRC‐FET protocols. By restricting the study population to FET cycles, we isolated the impact of the endometrial preparation method from the potential influence of the cryopreservation process. In this direct comparison, HRC‐FET was associated with a significantly higher risk of major complication compared to NC‐FET. These findings suggest that the adverse effects are inherently linked to the physiological milieu unique to HRC‐FET.

A fundamental limitation impeding clear clinical recommendations in ART has been the fragmented nature of existing scientific literature. Historically, studies assessing ART outcomes have often been constrained by small sample sizes or relied on heterogeneous cohorts, making it challenging to derive robust and generalizable conclusions, particularly for rare but serious complications. Furthermore, many investigations adopted simplistic binary comparisons, such as broadly comparing all FET cycles against Fresh ET, or focusing narrowly on comparing just two FET subgroups (HRC vs. NC). This practice frequently resulted in conflicting data that obscured the precise influence of differing hormonal environments on subsequent pregnancy outcomes. Our study overcomes these drawbacks by simultaneously comparing three major distinct clinical approaches (Fresh ET, HRC‐FET, and NC‐FET) within a large cohort, enabling a detailed assessment of risk associated with specific endometrial preparation methods. The ability to delineate the risks across Fresh ET, HRC, and NC‐FET allows us to better understand the potential associations between adverse outcomes and the presence or absence of key physiological conditions such as corpus luteum (CL) formation.

The most pronounced finding is that HRC‐FET was significantly associated with an increased risk of HDP, whereas no such association was observed for NC‐FET when compared with Fresh ET. Numerous prior studies have reported an increased risk of HDP following FET relative to Fresh ET [37, 38, 39]. However, several investigations have also found no statistically significant difference between the two approaches [4, 40, 41, 42]. The result of the present study robustly supports a growing body of evidence indicating that HRC‐FET cycles carry a higher risk of HDP [43, 44, 45, 46, 47]. In systematic reviews and meta‐analyses, HRC‐FET protocols have likewise been shown to be associated with a significantly increased risk of HDP, including pregnancy‐induced hypertension and preeclampsia, compared with NC‐FET [16, 17, 18]. These findings strongly suggest that the excess risk is attributable not to maternal characteristics or cryopreservation of embryos per se, but rather to the method of endometrial preparation at the time of ET. This increased risk is highly correlative with the key biological difference inherent in the HRC protocol characterized by the intentional suppression of ovulation and the subsequent absence of a CL. It has been postulated that the CL secretes vital vasoactive substances, such as relaxin and vascular endothelial growth factor, which are essential for appropriate cardiovascular adaptation and successful maternal vascular remodeling during early gestation [48, 49, 50]. The lack of these substances in HRC‐FET pregnancies is thought to lead to compromised maternal vascular health, resulting in the higher incidence of HDP and preeclampsia. The fact that our NC‐FET group, where a CL is formed, did not show a similarly elevated risk for HDP, provides critical, protocol‐specific validation for the CL hypothesis.

Our analysis further revealed that HRC‐FET showed a significant association with an increased risk of PAS, whereas this relationship was not evident for NC‐FET when compared with Fresh ET. In addition, we identified an increased risk of PPH due to uterine atony significantly associated with HRC‐FET, a pattern that was not observed for NC‐FET. Large‐scale registry studies conducted in Japan and meta‐analyses have reported that pregnancies following FET are associated with an increased risk of PAS and PPH compared with those following Fresh ET [12, 13, 39]. Furthermore, multiple studies have consistently demonstrated that, relative to NC‐FET, HRC‐FET is linked to a higher risk of PAS and PPH, thereby reinforcing the consistency of these findings [16, 43, 44, 45, 46, 51]. The increased risk of PPH found in our HRC‐FET group is supported by a previous morphological study that compared placentas from HRC‐FET cycles with NC‐FET and Fresh ET [52]. This study reported significantly higher levels of the fibrinoid layer and loss of decidua in the HRC‐FET group placentas, histological changes directly associated with increased bleeding during delivery. The mechanism linking the absence of the CL in HRC‐FET to PAS and PPH could be attributed to aberrant placentation, possibly through uncontrolled growth of the extravillous trophoblast into the myometrium, as well as increased vascular fragility due to suboptimal hormonal signaling [53, 54].

The broader comparison in ART literature between Fresh ET and FET remains nuanced. Several studies have demonstrated that, compared with Fresh ET, FET is associated with an increased risk of HDP, PAS, and PPH [11, 12, 13, 37, 38, 39]. However, the heterogeneity of previous studies often meant that the adverse effects of HRC‐FET were sometimes conflated with the overall characteristics of FET. Our study delineates these risks, suggesting that the investigated maternal and placental complications are primarily concentrated within the HRC protocol, whereas the outcomes for NC‐FET were broadly similar to those of Fresh ET, with differences that were unlikely to be clinically significant.

Regarding GDM, our data indicates no statistically significant difference in incidence among Fresh ET, HRC‐FET, and NC‐FET groups. Moreover, the RDs were small, and the aORs crossed 1. These findings are consistent with several previous reports, including recent large‐scale meta‐analyses, which have demonstrated no significant difference in the risk of GDM between FET and Fresh ET [4, 38, 39, 40, 41, 42]. Likewise, multiple systematic reviews have reported no significant difference in GDM risk between HRC‐FET and NC‐FET [16, 17, 18]. Similarly, we found no significant differences in the incidence of placenta previa across the three groups. Consistent with this, the RDs remained minimal, and the aORs had 95% CIs that included 1. While some studies have suggested that the incidence of placenta previa is lower in FET cycles compared with Fresh ET cycles [38, 39], recent meta‐analyses have found no significant difference in the risk of placenta previa between the two approaches [11, 12]. Furthermore, multiple systematic reviews have reported no significant difference in the risk of placenta previa between HRC‐FET and NC‐FET [16, 17, 18]. The consistency observed for GDM and placenta previa across protocols contrasts with the protocol‐specific patterns identified for HDP, PAS, and PPH due to uterine atony, suggesting that different maternal complications may be associated with distinct pathways related to ART.

In conclusion, this extensive, detailed analysis suggests that adverse maternal and placental outcomes, previously broadly attributed to FET, are largely concentrated within the HRC protocol. The significantly heightened risks observed for HDP, PAS, and PPH due to uterine atony associated with HRC‐FET highlight the need to consider these associations when selecting patients and providing risk counseling. Our data support the consideration of ovulatory‐based endometrial preparation strategies that are typically characterized by the presence of a CL, when medically and logistically feasible, given the associations observed with maternal and placental outcomes.

## Ethics Statement

The research protocol for this study was approved by the Institutional Ethics Committee of IVF Osaka Clinic. Given the study's retrospective design, the requirement for written informed consent was waived. Information regarding the purpose and conduct of the study was disclosed to all potential participants, and an opportunity to opt out was provided.

## Conflicts of Interest

The authors declare no conflicts of interest.

## Supporting information


**Table S1:** Multivariable Analysis for HDP: Results of Primary Causal Estimation and Sensitivity Analyses.

**Table S2:** Multivariable Analysis for GDM: Results of Primary Causal Estimation and Sensitivity Analyses.

**Table S3:** Multivariable Analysis for Placenta Previa: Results of Primary Causal Estimation and Sensitivity Analyses.

**Table S4:** Multivariable Analysis for PAS: Results of Primary Causal Estimation and Sensitivity Analyses.

**Table S5:** Multivariable Analysis for PPH due to Uterine Atony: Results of Primary Causal Estimation and Sensitivity Analyses.

**Table S6:** Multivariable Analysis for the Incidence of Any of the Five Complications: Results of Primary Causal Estimation and Sensitivity Analyses.

**Table S7:** Multivariable Analysis for the Incidence of Multiple Complications: Results of Primary Causal Estimation and Sensitivity Analyses.

## Data Availability

The data that support the findings of this study are available from the corresponding author upon reasonable request.
