# Supplementary material for: Hormone Replacement Cycle Frozen–Thawed Embryo Transfer Is Associated With Elevated Perinatal Risk Compared With Natural Ovulatory Cycle Frozen–Thawed and Fresh Embryo Transfers: Retrospective Analysis of 7,593 Live Birth Cycles
Source: Reprod Med Biol. 2026 Jul 6;25(1):e70072. doi: 10.1002/rmb2.70072 (PMC13334288; doi:10.1002/rmb2.70072)
Supplement: Supplementary file 1 — Table S1: Multivariable Analysis for HDP: Results of Primary Causal Estimation and Sensitivity Analyses. [file RMB2-25-e70072-s001.docx]

| Supplementary Table 1: Multivariable Analysis for HDP: Results of Primary Causal Estimation and Sensitivity Analyses | | | | |  |
| --- | --- | --- | --- | --- | --- |
|  |  |  |  |  |  |
|  | Primary Model | Maternal Age <36 | Maternal Age >35 | Direct Comparison |  |
| Covariate | aOR (95% CI) | aOR (95% CI) | aOR (95% CI) | aOR (95% CI) |  |
| Maternal age at transfer | 1.03 (1.01 to 1.05) | 1.00 (0.951 to 1.05) | 1.05 (1.00 to 1.10) | 1.03 (1.01 to 1.05) |  |
| BMI | 1.12 (1.09 to 1.14) | 1.11 (1.07 to 1.15) | 1.12 (1.09 to 1.16) | 1.13 (1.10 to 1.15) |  |
| History of delivery | 0.471 (0.382 to 0.580) | 0.518 (0.366 to 0.734) | 0.452 (0.348 to 0.586) | 0.459 (0.369 to 0.570) |  |
| Endometrial thickness at transfer | 0.976 (0.936 to 1.01) | 0.960 (0.902 to 1.02) | 0.987 (0.934 to 1.04) | 0.970 (0.928 to 1.01) |  |
| Endometrial preparation methods |  |  |  |  |  |
| Fresh ET | Reference | Reference | Reference | NA |  |
| HRC-FET | 1.96 (1.46 to 2.62) | 2.11 (1.31 to 3.39) | 1.83 (1.26 to 2.66) | 1.99 (1.61 to 2.47) |  |
| NC-FET | 0.983 (0.702 to 1.37) | 1.08 (0.626 to 1.86) | 0.906 (0.591 to 1.38) | Reference |  |
|  |  |  |  |  |  |
| The covariates for multivariable analysis included endometrial preparation methods, maternal age at transfer, BMI, history of delivery, and endometrial thickness at transfer. | | | | |  |
|  |  |  |  |  |  |
| HDP: hypertensive disorders of pregnancy, BMI: body mass index, HRC: hormone replacement cycle, NC: natural cycle, FET: frozen-thawed embryo transfer, aOR: adjusted odds ratio, CI: confidence interval | | | | |  |
|  |  |  |  |  |  |
