# Supplementary material for: Hormone Replacement Cycle Frozen–Thawed Embryo Transfer Is Associated With Elevated Perinatal Risk Compared With Natural Ovulatory Cycle Frozen–Thawed and Fresh Embryo Transfers: Retrospective Analysis of 7,593 Live Birth Cycles
Source: Reprod Med Biol. 2026 Jul 6;25(1):e70072. doi: 10.1002/rmb2.70072 (PMC13334288; doi:10.1002/rmb2.70072)
Supplement: Supplementary file 2 — Table S2: Multivariable Analysis for GDM: Results of Primary Causal Estimation and Sensitivity Analyses. [file RMB2-25-e70072-s004.docx]

| Supplementary Table 2: Multivariable Analysis for GDM: Results of Primary Causal Estimation and Sensitivity Analyses | | | | |  |
| --- | --- | --- | --- | --- | --- |
|  |  |  |  |  |  |
|  | Primary Model | Maternal Age <36 | Maternal Age >35 | Direct Comparison |  |
| Covariate | aOR (95% CI) | aOR (95% CI) | aOR (95% CI) | aOR (95% CI) |  |
| Maternal age at transfer | 1.08 (1.05 to 1.11) | 1.03 (0.962 to 1.10) | 1.05 (0.996 to 1.11) | 1.08 (1.05 to 1.11) |  |
| BMI | 1.15 (1.13 to 1.18) | 1.17 (1.12 to 1.22) | 1.14 (1.11 to 1.18) | 1.15 (1.12 to 1.18) |  |
| History of delivery | 0.870 (0.694 to 1.09) | 0.881 (0.569 to 1.36) | 0.861 (0.661 to 1.12) | 0.922 (0.729 to 1.16) |  |
| Endometrial thickness at transfer | 0.919 (0.873 to 0.969) | 0.908 (0.832 to 0.992) | 0.926 (0.868 to 0.989) | 0.921 (0.869 to 0.976) |  |
| Endometrial preparation methods |  |  |  |  |  |
| Fresh ET | Reference | Reference | Reference | NA |  |
| HRC-FET | 0.955 (0.702 to 1.29) | 1.24 (0.710 to 2.17) | 0.852 (0.589 to 1.23) | 1.18 (0.928 to 1.50) |  |
| NC-FET | 0.809 (0.571 to 1.14) | 0.873 (0.443 to 1.72) | 0.774 (0.513 to 1.16) | Reference |  |
|  |  |  |  |  |  |
| The covariates for multivariable analysis included endometrial preparation methods, maternal age at transfer, BMI, history of delivery, and endometrial thickness at transfer. | | | | |  |
|  |  |  |  |  |  |
| GDM: gestational diabetes mellitus, BMI: body mass index, HRC: hormone replacement cycle, NC: natural cycle, FET: frozen-thawed embryo transfer, aOR: adjusted odds ratio, CI: confidence interval | | | | |  |
|  |  |  |  |  |  |
