# Supplementary material for: Hormone Replacement Cycle Frozen–Thawed Embryo Transfer Is Associated With Elevated Perinatal Risk Compared With Natural Ovulatory Cycle Frozen–Thawed and Fresh Embryo Transfers: Retrospective Analysis of 7,593 Live Birth Cycles
Source: Reprod Med Biol. 2026 Jul 6;25(1):e70072. doi: 10.1002/rmb2.70072 (PMC13334288; doi:10.1002/rmb2.70072)
Supplement: Supplementary file 3 — Table S3: Multivariable Analysis for Placenta Previa: Results of Primary Causal Estimation and Sensitivity Analyses. [file RMB2-25-e70072-s006.docx]

| Supplementary Table 3: Multivariable Analysis for Placenta Previa: Results of Primary Causal Estimation and Sensitivity Analyses | | | | |  |
| --- | --- | --- | --- | --- | --- |
|  |  |  |  |  |  |
|  | Primary Model | Maternal Age <36 | Maternal Age >35 | Direct Comparison |  |
| Covariate | aOR (95% CI) | aOR (95% CI) | aOR (95% CI) | aOR (95% CI) |  |
| Maternal age at transfer | 1.03 (0.986 to 1.07) | 0.989 (0.889 to 1.10) | 1.05 (0.958 to 1.15) | 1.02 (0.975 to 1.06) |  |
| BMI | 1.02 (0.979 to 1.07) | 1.01 (0.940 to 1.09) | 1.03 (0.972 to 1.09) | 1.03 (0.981 to 1.08) |  |
| History of delivery | 1.06 (0.732 to 1.54) | 1.42 (0.795 to 2.55) | 0.906 (0.562 to 1.45) | 1.07 (0.732 to 1.57) |  |
| Endometrial thickness at transfer | 0.970 (0.885 to 1.06) | 1.01 (0.902 to 1.14) | 0.937 (0.819 to 1.07) | 0.944 (0.852 to 1.04) |  |
| Endometrial preparation methods |  |  |  |  |  |
| Fresh ET | Reference | Reference | Reference | NA |  |
| HRC-FET | 1.69 (0.881 to 3.25) | 2.48 (0.762 to 8.08) | 1.35 (0.614 to 2.97) | 1.07 (0.723 to 1.58) |  |
| NC-FET | 1.57 (0.772 to 3.21) | 2.24 (0.634 to 7.95) | 1.27 (0.537 to 3.01) | Reference |  |
|  |  |  |  |  |  |
| The covariates for multivariable analysis included endometrial preparation methods, maternal age at transfer, BMI, history of delivery, and endometrial thickness at transfer. | | | | |  |
|  |  |  |  |  |  |
| BMI: body mass index, HRC: hormone replacement cycle, NC: natural cycle, FET: frozen-thawed embryo transfer, aOR: adjusted odds ratio, CI: confidence interval | | | | |  |
|  |  |  |  |  |  |
