# Supplementary material for: Hormone Replacement Cycle Frozen–Thawed Embryo Transfer Is Associated With Elevated Perinatal Risk Compared With Natural Ovulatory Cycle Frozen–Thawed and Fresh Embryo Transfers: Retrospective Analysis of 7,593 Live Birth Cycles
Source: Reprod Med Biol. 2026 Jul 6;25(1):e70072. doi: 10.1002/rmb2.70072 (PMC13334288; doi:10.1002/rmb2.70072)
Supplement: Supplementary file 4 — Table S4: Multivariable Analysis for PAS: Results of Primary Causal Estimation and Sensitivity Analyses. [file RMB2-25-e70072-s003.docx]

| Supplementary Table 4: Multivariable Analysis for PAS: Results of Primary Causal Estimation and Sensitivity Analyses | | | | |  |
| --- | --- | --- | --- | --- | --- |
|  |  |  |  |  |  |
|  | Primary Model | Maternal Age <36 | Maternal Age >35 | Direct Comparison |  |
| Covariate | aOR (95% CI) | aOR (95% CI) | aOR (95% CI) | aOR (95% CI) |  |
| Maternal age at transfer | 1.00 (0.974 to 1.03) | 1.02 (0.947 to 1.10) | 1.00 (0.934 to 1.07) | 1.00 (0.970 to 1.03) |  |
| BMI | 0.970 (0.932 to 1.00) | 0.971 (0.918 to 1.02) | 0.970 (0.919 to 1.02) | 0.964 (0.928 to 1.00) |  |
| History of delivery | 1.43 (1.12 to 1.84) | 1.42 (0.964 to 2.09) | 1.45 (1.05 to 2.00) | 1.44 (1.12 to 1.86) |  |
| Endometrial thickness at transfer | 0.854 (0.795 to 0.917) | 0.884 (0.805 to 0.970) | 0.829 (0.745 to 0.923) | 0.851 (0.791 to 0.915) |  |
| Endometrial preparation methods |  |  |  |  |  |
| Fresh ET | Reference | Reference | Reference | NA |  |
| HRC-FET | 4.71 (2.41 to 9.21) | 21.0 (2.93 to 150) | 2.71 (1.32 to 5.59) | 3.23 (2.24 to 4.66) |  |
| NC-FET | 1.45 (0.695 to 3.05) | 6.00 (0.785 to 45.8) | 0.895 (0.393 to 2.03) | Reference |  |
|  |  |  |  |  |  |
| The covariates for multivariable analysis included endometrial preparation methods, maternal age at transfer, BMI, history of delivery, and endometrial thickness at transfer. | | | | |  |
|  |  |  |  |  |  |
| PAS: placenta accreta spectrum, BMI: body mass index, HRC: hormone replacement cycle, NC: natural cycle, FET: frozen-thawed embryo transfer, aOR: adjusted odds ratio, CI: confidence interval | | | | |  |
|  |  |  |  |  |  |
