# Supplementary material for: Hormone Replacement Cycle Frozen–Thawed Embryo Transfer Is Associated With Elevated Perinatal Risk Compared With Natural Ovulatory Cycle Frozen–Thawed and Fresh Embryo Transfers: Retrospective Analysis of 7,593 Live Birth Cycles
Source: Reprod Med Biol. 2026 Jul 6;25(1):e70072. doi: 10.1002/rmb2.70072 (PMC13334288; doi:10.1002/rmb2.70072)
Supplement: Supplementary file 5 — Table S5: Multivariable Analysis for PPH due to Uterine Atony: Results of Primary Causal Estimation and Sensitivity Analyses. [file RMB2-25-e70072-s005.docx]

| Supplementary Table 5: Multivariable Analysis for PPH due to Uterine Atony: Results of Primary Causal Estimation and Sensitivity Analyses | | | | |  |
| --- | --- | --- | --- | --- | --- |
|  |  |  |  |  |  |
|  |  |  |  |  |  |
|  | Primary Model | Maternal Age <36 | Maternal Age >35 | Direct Comparison |  |
| Covariate | aOR (95% CI) | aOR (95% CI) | aOR (95% CI) | aOR (95% CI) |  |
| Maternal age at transfer | 0.976 (0.955 to 0.998) | 0.965 (0.916 to 1.01) | 0.988 (0.934 to 1.04) | 0.980 (0.958 to 1.00) |  |
| BMI | 1.01 (0.993 to 1.04) | 1.02 (0.982 to 1.06) | 1.02 (0.984 to 1.05) | 1.01 (0.987 to 1.04) |  |
| History of delivery | 1.03 (0.850 to 1.24) | 0.741 (0.539 to 1.02) | 1.30 (1.01 to 1.67) | 1.06 (0.872 to 1.28) |  |
| Endometrial thickness at transfer | 0.986 (0.939 to 1.03) | 0.991 (0.925 to 1.06) | 0.981 (0.917 to 1.05) | 0.992 (0.943 to 1.04) |  |
| Endometrial preparation methods |  |  |  |  |  |
| Fresh ET | Reference | Reference | Reference | NA |  |
| HRC-FET | 3.29 (2.21 to 4.90) | 3.55 (2.00 to 6.29) | 3.04 (1.75 to 5.28) | 2.41 (1.88 to 3.08) |  |
| NC-FET | 1.37 (0.878 to 2.13) | 1.62 (0.859 to 3.08) | 1.15 (0.619 to 2.14) | Reference |  |
|  |  |  |  |  |  |
| The covariates for multivariable analysis included endometrial preparation methods, maternal age at transfer, BMI, history of delivery, and endometrial thickness at transfer. | | | | |  |
|  |  |  |  |  |  |
| PPH: postpartum hemorrhage, BMI: body mass index, HRC: hormone replacement cycle, NC: natural cycle, FET: frozen-thawed embryo transfer, aOR: adjusted odds ratio, CI: confidence interval | | | | |  |
|  |  |  |  |  |  |
