# Supplementary material for: Hormone Replacement Cycle Frozen–Thawed Embryo Transfer Is Associated With Elevated Perinatal Risk Compared With Natural Ovulatory Cycle Frozen–Thawed and Fresh Embryo Transfers: Retrospective Analysis of 7,593 Live Birth Cycles
Source: Reprod Med Biol. 2026 Jul 6;25(1):e70072. doi: 10.1002/rmb2.70072 (PMC13334288; doi:10.1002/rmb2.70072)
Supplement: Supplementary file 6 — Table S6: Multivariable Analysis for the Incidence of Any of the Five Complications: Results of Primary Causal Estimation and Sensitivity Analyses. [file RMB2-25-e70072-s007.docx]

| Supplementary Table 6: Multivariable Analysis for the Incidence of Any of the Five Complications: Results of Primary Causal Estimation and Sensitivity Analyses | | | | |  |
| --- | --- | --- | --- | --- | --- |
|  |  |  |  |  |  |
|  |  |  |  |  |  |
|  | Primary Model | Maternal Age <36 | Maternal Age >35 | Direct Comparison |  |
| Covariate | aOR (95% CI) | aOR (95% CI) | aOR (95% CI) | aOR (95% CI) |  |
| Maternal age at transfer | 1.02 (1.01 to 1.03) | 0.996 (0.962 to 1.03) | 1.03 (1.00 to 1.07) | 1.02 (1.00 to 1.03) |  |
| BMI | 1.09 (1.08 to 1.11) | 1.08 (1.05 to 1.11) | 1.10 (1.08 to 1.13) | 1.09 (1.07 to 1.11) |  |
| History of delivery | 0.834 (0.738 to 0.942) | 0.829 (0.678 to 1.01) | 0.848 (0.726 to 0.989) | 0.856 (0.755 to 0.972) |  |
| Endometrial thickness at transfer | 0.951 (0.924 to 0.979) | 0.952 (0.913 to 0.994) | 0.951 (0.914 to 0.989) | 0.951 (0.922 to 0.980) |  |
| Endometrial preparation methods |  |  |  |  |  |
| Fresh ET | Reference | Reference | Reference | NA |  |
| HRC-FET | 2.09 (1.72 to 2.53) | 3.05 (2.21 to 4.21) | 1.60 (1.25 to 2.04) | 1.95 (1.70 to 2.24) |  |
| NC-FET | 1.06 (0.860 to 1.32) | 1.41 (0.981 to 2.03) | 0.871 (0.663 to 1.14) | Reference |  |
|  |  |  |  |  |  |
| The covariates for multivariable analysis included endometrial preparation methods, maternal age at transfer, BMI, history of delivery, and endometrial thickness at transfer. | | | | |  |
|  |  |  |  |  |  |
| BMI: body mass index, HRC: hormone replacement cycle, NC: natural cycle, FET: frozen-thawed embryo transfer, aOR: adjusted odds ratio, CI: confidence interval | | | | |  |
|  |  |  |  |  |  |
