# Supplementary material for: Hormone Replacement Cycle Frozen–Thawed Embryo Transfer Is Associated With Elevated Perinatal Risk Compared With Natural Ovulatory Cycle Frozen–Thawed and Fresh Embryo Transfers: Retrospective Analysis of 7,593 Live Birth Cycles
Source: Reprod Med Biol. 2026 Jul 6;25(1):e70072. doi: 10.1002/rmb2.70072 (PMC13334288; doi:10.1002/rmb2.70072)
Supplement: Supplementary file 7 — Table S7: Multivariable Analysis for the Incidence of Multiple Complications: Results of Primary Causal Estimation and Sensitivity Analyses. [file RMB2-25-e70072-s002.docx]

| Supplementary Table 7: Multivariable Analysis for the Incidence of Multiple Complications: Results of Primary Causal Estimation and Sensitivity Analyses | | | | |  |
| --- | --- | --- | --- | --- | --- |
|  |  |  |  |  |  |
|  |  |  |  |  |  |
|  | Primary Model | Maternal Age <36 | Maternal Age >35 | Direct Comparison |  |
| Covariate | aOR (95% CI) | aOR (95% CI) | aOR (95% CI) | aOR (95% CI) |  |
| Maternal age at transfer | 1.03 (1.00 to 1.07) | 0.994 (0.920 to 1.07) | 1.04 (0.965 to 1.13) | 1.04 (1.00 to 1.07) |  |
| BMI | 1.10 (1.07 to 1.14) | 1.11 (1.05 to 1.17) | 1.10 (1.05 to 1.15) | 1.10 (1.06 to 1.14) |  |
| History of delivery | 0.671 (0.497 to 0.906) | 0.546 (0.313 to 0.954) | 0.739 (0.514 to 1.06) | 0.684 (0.504 to 0.928) |  |
| Endometrial thickness at transfer | 0.914 (0.851 to 0.981) | 0.920 (0.831 to 1.01) | 0.910 (0.825 to 1.00) | 0.901 (0.837 to 0.970) |  |
| Endometrial preparation methods |  |  |  |  |  |
| Fresh ET | Reference | Reference | Reference | NA |  |
| HRC-FET | 3.05 (1.77 to 5.25) | 2.35 (1.08 to 5.10) | 3.71 (1.72 to 8.00) | 3.09 (2.09 to 4.58) |  |
| NC-FET | 0.986 (0.519 to 1.87) | 0.960 (0.374 to 2.46) | 1.03 (0.427 to 2.50) | Reference |  |
|  |  |  |  |  |  |
| The covariates for multivariable analysis included endometrial preparation methods, maternal age at transfer, BMI, history of delivery, and endometrial thickness at transfer. | | | | |  |
|  |  |  |  |  |  |
| BMI: body mass index, HRC: hormone replacement cycle, NC: natural cycle, FET: frozen-thawed embryo transfer, aOR: adjusted odds ratio, CI: confidence interval | | | | |  |
|  |  |  |  |  |  |
